# Impact of sleep apnea severity and nocturnal hypoxemia on the triglyceride-rich lipoproteins particles: the ELSA-Brasil study

**DOI:** 10.1016/j.jlr.2026.101104

**Published:** 2026-07-14

**Authors:** Giuliano Generoso, Fernando Yue Cesena, Isabela M. Bensenor, Soraya Giatti, Barbara K. Parise, Ronaldo B. Santos, Raul D. Santos, Marcio S. Bittencourt, Paulo A. Lotufo, Luciano F. Drager

**Affiliations:** 1 Cardiology Center, Hospital Sirio-Libanes, Sao Paulo, Brazil; 2 Center for Clinical and Epidemiological Research, University Hospital, Sao Paulo, Brazil; 3 Instituto Dante Pazzanese de Cardiologia, São Paulo, São Paulo, Brazil; 4 Unidade de Hipertensão, Instituto do Coracao (InCor), Hospital das Clinicas, Faculdade de Medicina, Universidade de Sao Paulo, Sao Paulo, Brazil; 5 Unidade Clinica de Lipides, Instituto do Coracao (InCor), Hospital das Clinicas, Faculdade de Medicina, Universidade de Sao Paulo, Sao Paulo, Brazil; 6 Division of Cardiology, Department of Medicine, University of Pittsburgh, Pittsburgh, PA

**Keywords:** sleep apnea, hypoxemia, triglyceride-rich lipoproteins, triglycerides, NMR spectroscopy, lipoprotein subclasses, intermittent hypoxia, atherosclerosis

## Abstract

Intermittent hypoxia, a hallmark of obstructive sleep apnea (OSA), promotes impaired triglyceride-rich lipoprotein (TRL) clearance in mice models, but data in humans are unclear. We evaluated the association of total TRL-p, its subclasses and TRL mean size with OSA severity and nocturnal hypoxemia in participants with sleep study and lipid profile assessed by nuclear magnetic resonance spectroscopy. OSA was classified as none, mild, moderate, and severe. Sleep time under SpO_2_ <90% (TSA90) was categorized into tertiles and as 0%, >0–1%, 1%–10%, and ≥10%. Non-linear associations were tested using restricted cubic spline (RCS) regression. Models were adjusted for age, sex, race, hypertension, diabetes, smoking, low-density lipoprotein, and waist circumference. Thus, 2,397 participants (49 ± 8 years, 57% women) were included (33.5% moderate/severe OSA). After full adjustment, large/very large TRL-p were associated with mild (*P* = 0.016) and moderate (*P* = 0.032) but not with severe OSA. TRL mean size was associated with TSA90 tertiles 2–3, driven by large/very large TRL-p, irrespective of sex. RCS analysis confirmed a non-linear dose–response between TSA90 and large/very large TRL-p (*P* = 0.021) and TRL mean size (*P* = 0.011), characterized by a rise-and-plateau pattern with maximal effect at TSA90 ∼3% and progressive attenuation thereafter. Sensitivity analyses considering insulin resistance, lipid-lowering and anti-hypertensive drugs have shown similar results. Summary, large/very large TRL-p are independently associated with mild-moderate OSA and show a non-linear, rise-and-plateau dose–response with nocturnal hypoxemia, saturating at hypoxic burdens around 3% of sleep time—suggesting that even minimal hypoxic burden engages atherogenic TRL pathways and challenging AHI-based risk stratification.

## Graphical abstract

**Figure:**
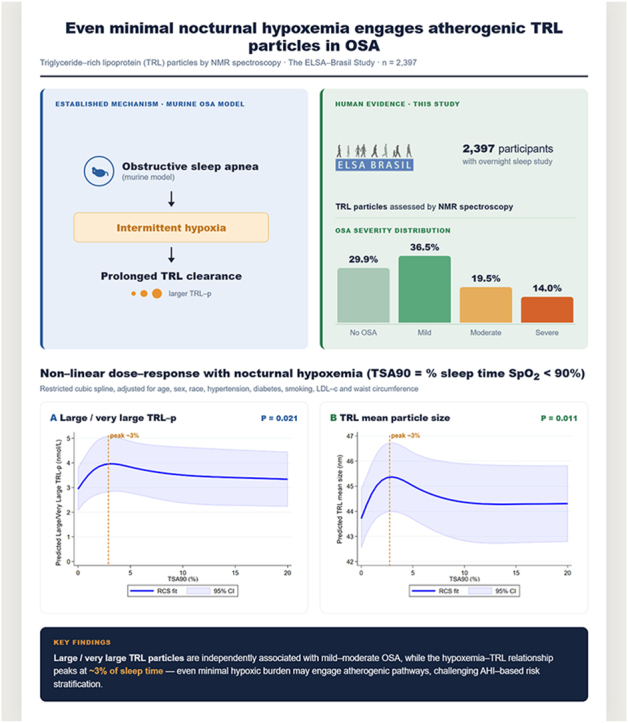

Obstructive sleep apnea (OSA) is strongly associated with metabolic syndrome (MS), changes in the lipid profile (1, 2, 3) and subclinical atherosclerosis (4, 5). Intermittent hypoxia, the key feature of OSA, has been extensively studied in animal models and may exacerbate MS components through increased sympathetic activation (6) and subsequent metabolic changes, resulting in higher fasting and postprandial levels of triglyceride-rich lipoproteins (TRL) due to impaired clearance (7).

In the TRL context, recent evidence is increasingly solidifying the link between TRL-cholesterol and atherosclerotic cardiovascular disease (8, 9). Further, clinical studies using nuclear magnetic resonance (NMR) spectroscopy, which enables precise quantification of lipoprotein particle size and concentration (10), showed that increased VLDL diameter—a proxy for larger TRL particle size—is positively associated with carotid plaques (11), myocardial infarction and ischemic stroke risk. These associations occur independent of traditional lipid measures (12). There is evidence that, per particle, TRL-p might be more atherogenic than LDL-p, driven by higher cholesterol content and direct uptake by macrophages leading to foam-cell formation and intimal cholesterol deposition; however, this remains debated, as other investigators have not found TRL particles to be more atherogenic than LDL particles. (13). However, the interplay between TRL-p (including its subclasses) and OSA in humans remains unknown.

Therefore, our study aimed to evaluate the association between total TRP-p and its subclasses (large/very large, medium and small/very small), TRL-p mean size with OSA according to its classification (mild, moderate and severe) and time during sleep under SpO2 <90%.

## Materials and methods

### Sample

ELSA-Brasil is a cohort with 15,105 active or retired Brazilian civil servants, aged 35–74 years from the six Brazilian cities (14). Exclusion criteria were recent pregnancy, cognitive impairment, and living in areas outside the study centers. The data collected involved extensive interviews, measurement of vital signs, 12-h nocturnal fasting blood collection and imaging exams (15) Race was self-reported as white, black, mixed brown, indigenous and Asian; smoking status, as never smoked, former smoker or current smoker; elevated blood pressure, as systolic blood pressure > 140 mmHg, diastolic blood pressure > 90 mmHg or in use of any anti-hypertensive drug; diabetes, as fasting blood glucose ≥ 126 mg/dl, HbA1c levels ≥ 6.5%, 2-h oral glucose tolerance test ≥ 200 mg or using anti-diabetic drug (16). Waist circumference was measured at the widest point of the greater trochanters (cm) with the participant standing upright and feet together. The occurrence of insomnia symptoms at any frequency was recorded as yes to examine the specific effect of insomnia symptoms over the last 30 nights, regardless of daytime sleepiness or sleep quantity. (17) For this study, we cross-sectionally analyzed the baseline assessment (2012–2014) of the São Paulo center participants who had complete lipid profile assessed by NMR and sleep study.

### Laboratory measurement

After blood collection, the samples were stored in freezers at −80°C and baseline laboratory determinations were performed by enzymatic and/or colorimetric assays. Further on, the whole lipid profile was measured by NMR spectroscopy (LipoProfile® 4 test spectra, LabCorp, Raleigh, NC, USA) (18). The method analyzed three main categories: (a) total cholesterol and fractions, triglycerides, apolipoproteins A1 and B (all in mg/dl); (b) HDL (in μmol/L), LDL and TRL particle number (in nmol/L); and (c) HDL-p, LDL-p and TRL-p mean size (in nm). Additionally, total TRL-p was divided into three subclasses: very large/large (50–240 nm diameter), medium (37–49 nm), and small/very small (24–36 nm).

### Sleep study

We used the Epworth Sleepiness Scale, validated for Portuguese language, to evaluate excessive daytime sleepiness. The scale ranged from 0 to 24, and we considered scores >10 as excessive daytime sleepiness (19). The overnight home sleep assessment was performed with a validated level 3 portable diagnostic device (Embletta Gold, Natus Medical Inc.), which has consistent sensitivity and specificity. A team of experts in sleep medicine manually scored the exams following the 2012 American Academy of Sleep Medicine criteria. Apnea was defined as a decrease of ≥ 90% in the airflow from the baseline value for ≥10 s. Hypopnea was defined as a drop of ≥ 30% in the airflow lasting at least 10 s combined with a 3% oxygen drop. Apneas were further classified as obstructive or central based on the presence or absence, respectively, of respiratory-related chest wall movement (20). The apnea-hypopnea index (AHI) was determined by the sum of apneas and hypopneas per hour.

We defined OSA in four different classifications: according to (a) OSA severity: none, mild (5–14 AHI), moderate (15–29 AHI), and severe (≥30 AHI); (b) a binary variable using AHI (lower than 15 – no OSA/mild vs. equal to or higher than 15 – moderate/severe OSA). Given the evidence linking hypoxic burden to cardiovascular disease (21), but the absence of consensus on optimal cutoffs for the percentage of sleep time with SpO_2_ <90% (TSA90), we examined two categorizations: (c) TSA90 = 0 versus tertiles when TSA90 > 0, and (d) clinically anchored categories (0, >0–1%, 1%–10%, ≥10%). The latter was exploratory and aimed at characterizing the dose–response between hypoxemic burden and TRL-p subclasses. To formally test non-linearity suggested by these categorical analyses, we additionally fitted restricted cubic spline (RCS) models, as described in the statistical analysis section.

### Statistical analysis

We presented continuous variables as mean and standard deviation for normal distribution and median (interquartile range) for non-normal variables. Normality was assessed using visual inspection of histograms. We compared the variables through ANOVA and unpaired *t* test in those with normal distributions and Kruskal–Wallis test for non-normal distributions. Additionally, we analyzed categorical variables by chi (2) test.

To assess the association between the lipid variable and each sleep disorder outcome, we constructed bivariate and multivariate regression models. Multiple regression models were built hierarchically, each model containing all covariates of the preceding one: Model 1 was adjusted for age, sex, and race; Model 2 added hypertension, diabetes, and smoking status; and Model 3 further added LDL-c and waist circumference. Thus, Model 3 included every covariate from Models 1 and 2 plus LDL-c and waist circumference. To formally test for non-linear associations suggested by the categorical analysis, we fitted restricted cubic spline (RCS) models with 4 knots placed at TSA90 values of 0%, 1%, 5%, and 15%, with adjustment for the same covariates as Model 3. Nonlinearity was tested by jointly testing the second and third spline coefficients.

### Sensitivity analysis

To assess whether insulin resistance accounted for the associations, Model 3 was refitted adding ln(HOMA-IR), restricted to participants not on antidiabetic treatment, with the diabetes term removed to avoid collinearity; those on antidiabetic treatment were analyzed separately with fasting plasma glucose replacing HOMA-IR, and the diabetes term was additionally replaced by ln(HbA1c) in the full sample. To determine whether the Model 3 attenuation reflected central adiposity, LDL-c, or both, waist circumference and LDL-c were added separately to Model 2. To exclude pharmacological effects on lipids, the fully adjusted analysis was repeated in participants not using lipid-lowering drugs and, separately, in those not using beta-blockers or thiazide diuretics. Finally, to assess effect modification by sex, baseline characteristics were described by sex and the fully adjusted model for TSA90 tertiles was refitted separately in men and women. We defined the *P*-values as 2-sided with an α of 0.05. All the analyses were performed in Stata 17.0 (StataCorp LLC).

### Ethical aspects

The ELSA-Brasil cohort was conducted in strict compliance with the ethical guidelines regulating research involving humans in Brazil. The study protocol was reviewed and approved by the National Research Ethics Commission (CONEP) and by the Institutional Review Boards of each of the six co-participating research and higher education institutions (USP, Fiocruz, UFMG, UFBA, UFRGS, and UFES). All volunteers agreed to participate in the study by signing a written Informed Consent Form obtained prior to any examination or interview. Participant anonymity, as well as the privacy and confidentiality of all stored clinical, epidemiological, and biological data, were strictly maintained, fully respecting the regulations of Resolution No. 466/2012 of the Brazilian National Health Council and the principles of the Declaration of Helsinki.

## Results

A total of 2,397 participants (49 ± 8 years old, 43% male) were included in the analysis. As expected, those who had moderate/severe OSA (AHI ≥15) accounted for 803 (33.5%) of the sample and, compared with participants with an AHI <15, exhibited a substantially different baseline profile. They were older and more frequently male, and carried a higher burden of cardiometabolic comorbidities, including hypertension and diabetes mellitus. This was paralleled by greater adiposity (higher body mass index and waist circumference) and a more insulin-resistant phenotype, with higher fasting insulin, HOMA-IR, and fasting plasma glucose. Their lipid profile was also more atherogenic, with higher triglycerides, total cholesterol, LDL-cholesterol, and apolipoprotein B, together with lower HDL-cholesterol and apolipoprotein A-1. Accordingly, total TRL-p and all TRL subclasses were higher in the moderate/severe OSA group, most markedly the large/very large TRL-p subclass (Table 1). While 38.7% of the participants had an excessive daytime sleepiness score and half of them reported sleep time under 7 h per night, only 5.6% had an insomnia diagnosis (Table 2).

**Table 1:** Baseline characteristics of the studied sample

	Total (n = 2,397)	AHI <15 (n = 1,594)	AHI ≥15 (n = 803)	*P*-value
Age (years)	49.3 (8.3)	48.2 (8.0)	51.6 (8.5)	<0.001
Female sex	1,357 (56.6%)	1,013 (63.6%)	344 (42.8%)	
Race/ethnicity, n (%)				0.22
Black	291 (12.3%)	201 (12.7%)	90 (11.3%)	
Mixed Brown	489 (20.6%)	309 (19.5%)	180 (22.7%)	
White	1,454 (61.2%)	985 (62.3%)	469 (59.1%)	
Asian	122 (5.1%)	75 (4.7%)	47 (5.9%)	
Indigenous	19 (0.8%)	12 (0.8%)	7 (0.9%)	
Smoking status, n (%)				0.006
Never	1,331 (55.5%)	913 (57.3%)	418 (52.1%)	
Former	725 (30.2%)	448 (28.1%)	277 (34.5%)	
Current	341 (14.2%)	233 (14.6%)	108 (13.4%)	
Hypertension, n (%)	642 (26.8%)	312 (19.6%)	330 (41.1%)	<0.001
Diabetes mellitus, n (%)	384 (16.0%)	187 (11.7%)	197 (24.5%)	
Insulin (μUI/ml)	6.3 (3.7–10.6)	5.5 (3.2–9.4)	8.2 (5.2–13.1)	
HOMA-IR	1.63 (0.93–2.96)	1.41 (0.81–2.47)	2.24 (1.35–3.65)	
Body mass index (kg/m^2^)	27.0 (4.8)	25.8 (4.2)	29.4 (5.0)	
Waist circumference (cm)	89.0 (12.3)	85.5 (10.9)	96.0 (11.8)	
Fasting plasma glucose (mg/dl)	108.4 (23.4)	105.3 (18.5)	114.5 (29.9)	
Total cholesterol (mg/dl)	197.6 (35.7)	195.0 (34.6)	202.6 (37.4)	
Triglycerides (mg/dl)	106.0 (74.0–152.0)	98.0 (69.0–139.0)	123.0 (87.0–177.0)	
HDL-cholesterol (mg/dl)	52.9 (11.4)	53.7 (11.4)	51.3 (11.3)	
LDL-cholesterol (mg/dl)	116.7 (30.2)	114.9 (29.4)	120.2 (31.3)	
Apolipoprotein B (mg/dl)	93.7 (23.8)	91.4 (23.0)	98.2 (24.9)	
Apolipoprotein A-1 (mg/dl)	137.9 (23.1)	138.7 (22.7)	136.2 (23.8)	0.014
Total TRL-p (nmol/L)	148.9 (73)	141.6 (69.1)	163.3 (78.1)	<0.001
Large/very large TRL-p (nmol/L)	1.6 (0.2–6.3)	0.9 (0.2–5.0)	3.3 (0.6–8.2)	
Medium TRL-p (nmol/L)	21.1 (18.0)	19.5 (16.9)	24.1 (19.8)	
Small/very small TRL-p (nmol/L)	123.5 (61.0)	118.4 (57.4)	133.5 (66.3)	

Data are presented as mean (SD) or median (IQR) for continuous measures, and n (%) for categorical measures.

HDL, high-density lipoprotein; AHI, apnea-hypopnea index; LDL, low-density lipoprotein.

**Table 2:** Variables assessed by the sleep study

	Total (N = 2,397)
Excessive daytime sleepiness (Epworth ≥10), n (%)	927 (38.7%)
Self-reported sleep duration, n (%)	
<5 h	26 (1.1%)
5–6 h	85 (3.6%)
6–7 h	1,088 (46.2%)
7–8 h	1,092 (46.3%)
>8 h	66 (2.8%)
Total polygraphy recording time (minutes)	423.5 (76.7)
Apnea-hypopnea index (events/hour)	9.9 (4.5–19.7)
Baseline peripheral oxygen saturation (%)	94.4 (93.2–95.5)
Mean peripheral oxygen saturation (%)	94.3 (93.1–95.3)
Minimum peripheral oxygen saturation (%)	86.0 (82.0–89.0)
Time with SpO2 below 90% tertiles, (% of time)	
No nocturnal hypoxemia (n = 735)	0
Tertile 1 (n = 554)	0.2 (0.1–0.3)
Tertile 2 (n = 559)	1.4 (0.9–2.1)
Tertile 3 (n = 547)	8.8 (4.9–17.0)
OSA severity category, n (%)	
No OSA (AHI <5)	717 (29.9%)
Mild OSA (AHI 5–14.9)	875 (36.5%)
Moderate OSA (AHI 15–29.9)	468 (19.5%)
Severe OSA (AHI ≥30)	335 (14.0%)
Insomnia diagnosis, n (%)	135 (5.6%)

Data are presented as mean (SD) or median (IQR) for continuous measures, and n (%) for categorical measures.

OSA, obstructive sleep apnea.

TSA90 tertile cutoffs were defined as follows: tertile 1 (0.1%–0.5%), tertile 2 (0.6%–3.1%), and tertile 3 (≥3.2%). Although the median percentage of time with SpO_2_ <90% differed substantially between tertile 2 (1.4%) and tertile 3 (8.8%), the clinical profile of participants in these tertiles was similar (Supplemental Table S1). Therefore, we further combined tertiles 2 and 3 into a single category and compared them with participants without nocturnal hypoxemia (TSA90 = 0/tertile 1).

### OSA classification and triglyceride-rich lipoproteins

In the linear regression analysis, we found a positive association between TRL-c, TRL-p and TRL mean size and all OSA classification when adjusted for epidemiological and risk factor covariates (Model 1 and Model 2), but this finding was attenuated to non-significance when adjusted for LDL-c and waist circumference (Model 3, *P =* NS). However, while medium and small/very small particles showed no association with any classification (*P =* NS), large/very large TRL-p subclass was independently associated with mild (β = 0.74 [95% CI, 0.14–1.35]) and moderate (β = 0.82 [95% CI, 0.07–1.58]) OSA diagnosis even after full adjustment (Table 3). When stratified by AHI cutoff of 15, we found no association in all models (all *P =* NS, Supplemental Table S2).

**Table 3:** Association between OSA diagnosis and triglyceride-rich lipoprotein profile and its subclasses

	Crude β(95% CI)	Model 1 β(95% CI)	Model 2 β(95% CI)	Model 3 β(95% CI)
TRL-cholesterol (mg/dl)				
No OSA	Reference			
Mild	4.24 (2.89; 5.58)	3.29 (1.94; 4.64)	2.92 (1.58; 4.26)	1.05 (−0.21; 2.31)
Moderate	6.52 (4.93; 8.12)	4.90 (3.27; 6.52)	4.19 (2.56; 5.82)	1.37 (−0.20; 2.94)
Severe	7.83 (6.05; 9.60)	5.67 (3.85; 7.48)	4.67 (2.84; 6.51)	1.32 (−0.47; 3.11)
Total TRL-P (nmol/L)				
No OSA	Reference			
Mild	19.54 (12.45; 26.63)	14.70 (7.57; 21.84)	13.29 (6.16; 20.42)	3.50 (−2.93; 9.93)
Moderate	29.48 (21.11; 37.84)	21.32 (12.72; 29.93)	18.63 (9.97; 27.30)	4.24 (−3.77; 12.26)
Severe	37.01 (27.69; 46.33)	26.55 (16.94; 36.16)	22.61 (12.85; 32.36)	5.78 (−3.36; 14.91)
Large/very large TRL-P (nmol/L)				
No OSA	Reference			
Mild	1.98 (1.37; 2.58)	1.70 (1.09; 2.31)	1.46 (0.85; 2.06)	0.74 (0.14; 1.35)^a^
Moderate	3.04 (2.32; 3.75)	2.57 (1.83; 3.31)	2.02 (1.29; 2.76)	0.82 (0.07; 1.58)^b^
Severe	3.55 (2.76; 4.35)	2.91 (2.08; 3.73)	2.21 (1.39; 3.04)	0.68 (−0.18; 1.54)
Medium TRL-P (nmol/L)				
No OSA	Reference			
Mild	4.26 (2.50; 6.01)	3.19 (1.45; 4.93)	2.75 (1.02; 4.49)	1.67 (−0.10; 3.44)
Moderate	6.35 (4.28; 8.43)	4.53 (2.43; 6.62)	3.74 (1.63; 5.84)	2.02 (−0.18; 4.23)
Severe	7.81 (5.50; 10.12)	5.27 (2.93; 7.61)	4.18 (1.81; 6.55)	2.06 (−0.46; 4.58)
Small/very small TRL-P (nmol/L)				
No OSA	Reference			
Mild	13.31 (7.36; 19.26)	9.81 (3.78; 15.84)	9.07 (3.03; 15.12)	1.08 (−4.14; 6.30)
Moderate	20.09 (13.07; 27.12)	14.23 (6.96; 21.50)	12.88 (5.53; 20.22)	1.40 (−5.10; 7.90)
Severe	25.64 (17.82; 33.47)	18.37 (10.25; 26.50)	16.21 (7.94; 24.48)	3.03 (−4.38; 10.45)
TRL mean size (nm)				
No OSA	Reference			
Mild	2.39 (1.53; 3.26)	1.98 (1.11; 2.86)	1.66 (0.79; 2.52)	0.34 (−0.50; 1.19)
Moderate	4.50 (3.48; 5.53)	3.86 (2.80; 4.92)	3.06 (2.01; 4.12)	0.79 (−0.27; 1.84)
Severe	5.98 (4.84; 7.12)	5.12 (3.94; 6.30)	4.02 (2.83; 5.20)	1.09 (−0.11; 2.29)

Model 1 was adjusted for sex, race, and age; Model 2, hypertension, diabetes, and smoking status (never, former, and current smoker); and Model 3, LDL-c and waist circumference. OSA means obstructive sleep apnea; TRL-c, triglyceride-rich lipoprotein-cholesterol; TRL-p, triglyceride-rich lipoprotein-particle number; and TRL, triglyceride-rich lipoprotein.

a *P* = 0.016.

b *P* = 0.032.

### Nocturnal hypoxemia

Although we found a positive association between TRL-c and TRL-p and all TSA90 tertiles in the crude analysis, the association was attenuated after adjustment for most risk biomarkers, and it was no longer significant after additionally controlling for waist circumference and LDL-c (*P* = NS). On the other hand, TRL mean size remained associated with both tertile 2 and 3 even after full adjustment (*P =* 0.009 and *P =* 0.025, respectively). This was driven by the large/very large TRL-p subfraction (β = 0.92 [95% CI, 0.23–1.60] and 0.80 [95% CI, 0.07–1.54] for tertiles 2 and 3, Table 4) and medium TRL-p for tertile 2 (β = 2.32 [95%CI, 0.32–4.33]).

**Table 4:** Association between rate of sleep time under SpO2 < 90% (tertiles) and triglyceride-rich lipoprotein profile and its subclasses

	TSA90	Crude β (95% CI)	Model 1 β (95% CI)	Model 2 β (95% CI)	Model 3 β (95% CI)
TRL-cholesterol (mg/dl)	Zero	Reference			
	Tertile 1	3.15 (1.63; 4.66)	2.28 (0.78; 3.77)	1.90 (0.42; 3.39)	0.76 (−0.61; 2.12)
	Tertile 2	4.94 (3.42; 6.45)	3.94 (2.43; 5.45)	3.42 (1.91; 4.92)	1.15 (−0.28; 2.57)
	Tertile 3	5.99 (4.47; 7.51)	4.57 (3.01; 6.12)	3.66 (2.10; 5.23)	0.83 (−0.70; 2.36)
Total TRL-P (nmol/L)	Zero	Reference			
	Tertile 1	15.17 (7.22; 23.13)	10.90 (3.00; 18.80)	9.41 (1.52; 17.29)	3.36 (−3.59; 10.32)
	Tertile 2	22.13 (14.20; 30.07)	16.61 (8.62; 24.60)	14.57 (6.56; 22.57)	3.02 (−4.25; 10.29)
	Tertile 3	29.54 (21.56; 37.52)	21.98 (13.77; 30.19)	18.41 (10.10; 26.73)	4.76 (−3.05; 12.57)
Large/very large TRL-P (nmol/L)	Zero	Reference			
	Tertile 1	1.22 (0.54; 1.90)	0.99 (0.32; 1.67)	0.78 (0.12; 1.45)	0.41 (−0.25; 1.06)
	Tertile 2	2.41 (1.73; 3.08)	2.16 (1.47; 2.84)	1.82 (1.14; 2.50)	0.92 (0.23; 1.60)^c^
	Tertile 3	3.13 (2.45; 3.81)	2.78 (2.08; 3.49)	2.19 (1.49; 2.90)	0.80 (0.07; 1.54)^d^
Medium TRL-P (nmol/L)	Zero	Reference			
	Tertile 1	3.48 (1.51; 5.45)	2.58 (0.66; 4.51)	2.13 (0.21; 4.04)	1.47 (−0.45; 3.38)
	Tertile 2	5.18 (3.21; 7.15)	4.40 (2.45; 6.35)	3.79 (1.84; 5.73)	2.32 (0.32; 4.33)
	Tertile 3	4.65 (2.67; 6.63)	3.39 (1.39; 5.39)	2.34 (0.32; 4.36)	0.25 (−1.90; 2.40)
Small/very small TRL-P (nmol/L)	Zero	Reference			
	Tertile 1	10.47 (3.81; 17.14)	7.33 (0.66; 13.99)	6.50 (−0.18; 13.18)	1.49 (−4.15; 7.13)
	Tertile 2	14.55 (7.90; 21.20)	10.05 (3.30; 16.80)	8.96 (2.18; 15.74)	−0.22 (−6.12; 5.68)
	Tertile 3	21.76 (15.07; 28.45)	15.80 (8.87; 22.74)	13.88 (6.84; 20.92)	3.71 (−2.63; 10.05)
TRL size (nm)	Zero	Reference			
	Tertile 1	1.98 (1.01; 2.95)	1.70 (0.73; 2.67)	1.39 (0.44; 2.35)	0.71 (−0.21; 1.62)
	Tertile 2	3.86 (2.89; 4.83)	3.48 (2.50; 4.47)	2.97 (2.00; 3.94)	1.27 (0.32; 2.23)^a^
	Tertile 3	5.30 (4.33; 6.28)	4.72 (3.71; 5.73)	3.81 (2.81; 4.82)	1.17 (0.15; 2.20)^b^

TSA90 tertile cutoffs were defined as follows: tertile 1 (0.1%–0.5%), tertile 2 (0.6%–3.1%), and tertile 3 (≥3.2%).

Model 1 was adjusted for sex, race, and age; Model 2, hypertension, diabetes, and smoking status (never, former, and current smoker); and Model 3, LDL-c and waist circumference. TSA90 means percentage of time under SpO2 <90%; TRL-c, triglyceride-rich lipoprotein-cholesterol; TRL-p, triglyceride-rich lipoprotein-particle number; and TRL, triglyceride-rich lipoprotein.

a *P* = 0.009.

b *P* = 0.033.

c *P* = 0.009.

d *P* = 0.025.

**Fig. 1:**
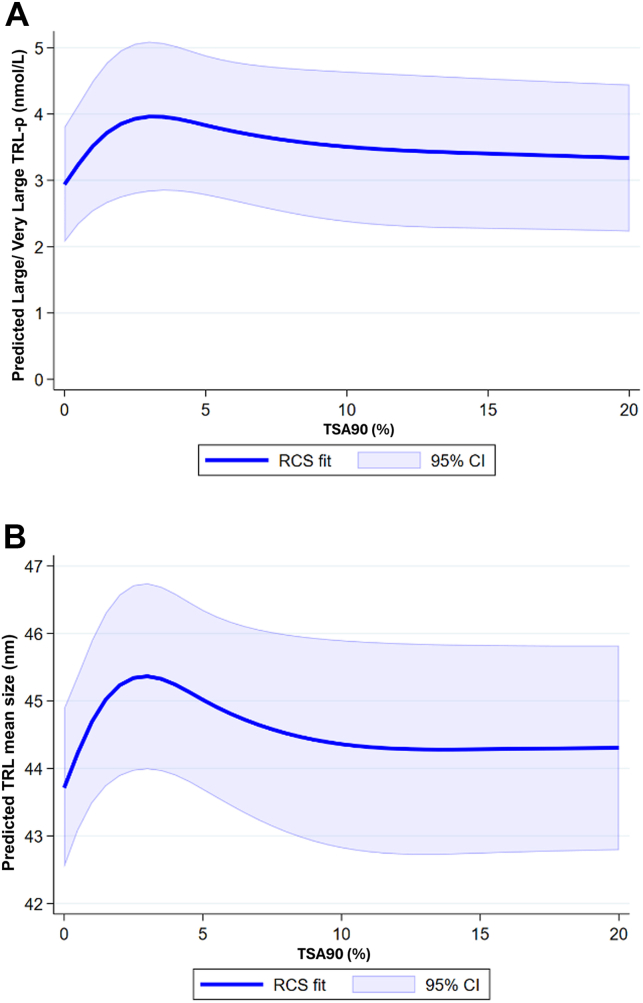
Restricted cubic spline regression curves showing the non-linear association between nocturnal hypoxemic burden (TSA90) and triglyceride-rich lipoprotein particle subclasses. Panel A (Large/Very Large TRL-p): Demonstrates a steep increase in large particle concentration from TSA90 = 0% to approximately 3%, followed by progressive attenuation at higher hypoxic burdens, with stabilization (plateau) beyond 10% TSA90. Non-linearity test: F(22,357) = 3.87, *P* = 0.021. Panel B (TRL mean size): Shows a similar rise-and-plateau pattern, with peak effect at ∼3% TSA90 and progressive attenuation thereafter. The effect magnitude is larger than for large TRL-p, consistent with the dominance of large particles in determining mean particle size. Non-linearity test: F(22,357) = 4.56, *P* = 0.011. Models are fully adjusted for age, sex, race, hypertension, diabetes, smoking status, LDL-cholesterol, and waist circumference. Shaded areas represent 95% confidence intervals around the fitted RCS curves. Knots placed at TSA90 = 0%, 1%, 5%, and 15%.

To formally test the non-linear dose–response pattern suggested by the categorical analysis, we fitted restricted cubic spline models with the same covariate adjustment as Model 3. RCS analysis confirmed significant non-linearity for large/very large TRL-p (F = 3.87, *P* = 0.021) and TRL mean size (F = 4.56, *P* = 0.011), characterized by a rise-and-plateau pattern: a steep increase from TSA90 = 0% to approximately 3%, followed by progressive attenuation and stabilization at higher hypoxic burdens. Finally, no non-linear association was observed for medium TRL-p (F = 0.76, *P* = 0.468), reinforcing the specificity of the hypoxemia effect to the large/very large TRL-p subclass (Table 5 and Fig. 1). To identify which covariate drove the Model 3 attenuation, waist circumference and LDL-c were added separately to Model: LDL-c alone weakly changed the estimates, whereas WC alone reproduced nearly the entire attenuation, indicating that central adiposity, not LDL-c, accounted for most of it, with a residual adiposity-independent association persisting for tertiles 2 and 3 (Supplemental Table S10).

**Table 5:** Restricted Cubic Spline Analysis: Non-linear Association between TSA90 and Triglyceride-Rich Lipoprotein Particles

	R^2^	Model F-test	Model *P*-value	Non-linearity F-test	Non-linearity *P*-value
Large/Very Large TRL-p (nmol/L)	0.183	2.78	0.040	3.87	0.021
TRL mean size (nm)	0.197	3.05	0.028	4.56	0.011
Medium TRL-p (nmol/L)	0.11	2.19	0.087	0.76	0.468

Restricted cubic spline (RCS) models were fitted with 4 knots at fixed TSA90 values of 0%, 1%, 5%, and 15% (percentage of sleep time with SpO_2_ <90%). Outcomes were van der Waerden (inverse-normal rank) transformed. Models were adjusted for sex, race, age, hypertension, diabetes, smoking status (never, former, current), LDL-c, and waist circumference (n = 2,397). The TSA90 spline association tests the joint contribution of the three spline terms (3 df); non-linearity was tested by jointly testing the second and third spline coefficients (TSA90_spl2 = TSA90_spl3 = 0; 2 df). *R*^2^ is the proportion of variance explained by the model; F is the F-statistic with degrees of freedom in parentheses.

### Sensitivity analysis

In participants not receiving antidiabetic treatment (n = 2,208), additional adjustment for ln(HOMA-IR) the association of nocturnal hypoxemia with large/very large TRL-p or TRL mean size increased monotonically across TSA90 tertiles, with significant linear trends (tertile 3, β = 0.80 [95% CI, 0.06–1.54], *P* for trend = 0.029; and β = 1.13 [0.05–2.21], *P* for trend = 0.028, respectively), whereas medium TRL-p showed no association (*P* = NS). Further, ln(HOMA-IR) was the strongest independent correlate of all three measures (β = 1.66, 2.40, and 3.65, respectively; all *P* < 0.001) but did not eliminate the hypoxemia associations. Consistent with the analysis including all samples, the largest increment occurred at low hypoxic burden (Supplemental Table S3). Finally, the analysis in subgroup participants on treatment for diabetes (n = 164) was underpowered and showed no consistent association (Supplemental Table S4). Additionally, findings were concordant when the diabetes term was replaced by continuous ln(HbA1c) in the full sample: large/very large TRL-p and TRL mean size remained associated with TSA90 tertiles 2 and 3 (*P* for trend = 0.017 and 0.018, respectively), with ln(HbA1c) an independent correlate of all TRL measures (Supplemental Table S5).

The associations were robust to medication use: the pattern of associations was similar in participants not taking lipid-lowering drugs (n = 2,107; Supplemental Table S6) and in those not using beta-blockers or thiazide diuretics (n = 1,952; Supplemental Table S7). In sex-stratified analyses, large/very large TRL-p and TRL mean size were positive across the upper hypoxemia tertiles in both men (n = 1,040) and women (n = 1,357), with medium TRL-p unassociated; confidence intervals overlapped substantially with no reversal of direction, indicating no meaningful effect modification by sex (Supplemental Tables S8 and S9).

## Discussion

In a large study of adults, our results showed a consistent association of large/very large TRL particles with mild and moderate OSA, which was not observed in other subclasses. Furthermore, we identified a significant non-linear association with nocturnal hypoxemia, assessed by the percentage of sleep time with SpO_2_ <90% (TSA90), characterized by a *rise-and-plateau* pattern with peak effect at low hypoxic burdens. These findings extend previous animal and human evidence linking OSA's hallmark feature — intermittent hypoxia — to atherogenic lipoproteins (6, 22, 23) and reinforce the role of OSA in promoting metabolic impairment, paving the way for increasing cardiovascular risk independently of potential confounding factors (24).

Recently, a case-control study found higher remnant cholesterol levels in patients with impaired sleep parameters measures (25). Notably, the association was confined to mild and moderate OSA, but absent in severe cases. In murine OSA models, IH inhibited LPL (lipoprotein lipase) and prolonged TRL clearance, predicting elevated circulating TRL particles. Our data extend this mechanism to humans by showing the increased large/very large TRL-p population most directly affected by impaired LPL hydrolysis—in those with mild-moderate OSA (22). Further, this selective pattern might reflect early-stage metabolic dysregulation, in which acute IH triggers lipolysis and TRL elevation before adaptive responses (as hypoxia-induced AMPK activation (26)) might attenuate effects in severe OSA. Further studies addressing this important issue are warranted. To note, this delayed clearance likely acts alongside hepatic VLDL/apoB overproduction and remnant remodeling rather than in isolation, so that LDL-c, which reflects cholesterol mass rather than particle number, is an insensitive marker of these upstream changes.

Additionally, we investigated the dose–response between hypoxic burden and TRL subclasses. Restricted cubic spline analysis formally confirmed a significant non-linear association for both large/very large TRL-p (P for non-linearity = 0.021) and TRL mean size (*P* = 0.011), characterized by a steep increase from TSA90 = 0% to approximately 3%, followed by progressive attenuation and stabilization at higher hypoxic burdens. Importantly, medium TRL-p showed no significant non-linear association (*P* = 0.468), reinforcing the specificity of the hypoxemia effect to the large TRL-p subclass. To our knowledge, this is the first demonstration of a formally tested non-linear dose–response relationship between nocturnal hypoxemia and TRL metabolism. The early saturation of the effect—with maximal impact at hypoxic burdens as low as 3% of total sleep time—challenges the conventional assumption of a monotonic dose-dependent relationship in OSA-related dyslipidemia and suggests that even minimal hypoxemic exposure may engage the relevant pathophysiological pathways. Mechanistically, brief IH may activate hypoxia-inducible factor-1 (HIF-1) in adipocytes (23), upregulating Angptl4 and inhibiting LPL, thereby delaying TRL clearance. (22) Prolonged hypoxemia, conversely, might overlap with chronic conditions (e.g., pulmonary diseases causing basal hypoxia), diluting associations, or trigger counter-regulatory pathways like enhanced lipid peroxidation resistance. The clinical implication is potentially important: TRL-related cardiovascular risk may not scale linearly with OSA severity as measured by AHI or TSA90, and patients with even modest hypoxemic burden may warrant metabolic surveillance.

The metabolic context of these associations deserves comment. In the fully adjusted models, most of the attenuation of the hypoxemia–TRL association was attributable to central adiposity rather than to LDL-c: adding waist circumference alone reproduced nearly the entire attenuation, whereas adding LDL-c alone left the estimates essentially unchanged. This is consistent with obesity acting as a central node linking intermittent hypoxia to atherogenic lipoproteins. (3) Nevertheless, a residual association of nocturnal hypoxemia with large/very large TRL-p and TRL mean size persisted after adjustment for waist circumference, indicating an adiposity-independent component. Insulin resistance appeared to act in parallel rather than as the explanation: although ln(HOMA-IR) was the strongest independent correlate of all TRL measures, its inclusion did not abolish the hypoxemia associations, which retained a significant linear trend across TSA90 tertiles. Together, these analyses suggest that nocturnal hypoxemia, central adiposity, and insulin resistance are overlapping but non-redundant correlates of larger TRL particles, and that hypoxemia contributes to atherogenic TRL beyond what is captured by adiposity and insulin resistance alone.

Recent studies have found a robust causal association of TRL with inflammation (13). Previously we also observed an independent association of the larger TRL-p with GlycA and leukocyte counts (27). While this fact could confound OSA-associated risk factors analyses, the findings of independent association indicate a potential contribution of intermittent hypoxia to low-grade inflammation and possibly atherogenesis. Although the relative per-particle atherogenicity of TRL-p versus LDL-p remains debated, some evidence suggests it might be up to four-fold greater; (13) if so, even the modest increases observed in our study could merit attention and may contribute to the heightened cardiovascular morbidity associated with OSA.

Of importance, the reversibility of these findings with OSA treatment warrants exploration. Continuous positive airway pressure (CPAP) has been shown to improve several cardiometabolic features of OSA, including insulin sensitivity (28), blood pressure (29) and endothelial dysfunction (30), with recent evidence suggesting a possible reduction in mortality (31). Furthermore, studies found that CPAP therapy significantly reduced postprandial triglycerides and total cholesterol levels in patients with severe OSA (32, 33). Another trial showed that weight loss, alone or combined with CPAP, was superior to CPAP monotherapy for improving the lipid profile and reducing triglycerides (34). Together with prior studies, our findings may point to the potential need for earlier metabolic surveillance and lifestyle intervention in patients with intermittent nocturnal hypoxemia, rather than a treatment approach based solely on AHI severity.

These findings were robust to several potential sources of bias. The associations of nocturnal hypoxemia with large/very large TRL-p and TRL mean size were preserved after excluding participants using lipid-lowering drugs and, separately, those using beta-blockers or thiazide diuretics (drugs that can raise triglycerides), indicating that the results were not driven by pharmacological effects on the lipid profile. Moreover, the direction and pattern of the associations were consistent in men and women, with no evidence of meaningful effect modification by sex, supporting their generalizability across both sexes.

The current investigation has limitations. As a cross-sectional analysis, we cannot infer causality. Sleep data were available only from a subgroup of participants from the São Paulo center. The non-linear pattern at higher TSA90 values may also be affected by limited statistical power in the upper extreme of the hypoxemic distribution. Notably, participants with OSA differed from those without OSA across a broad range of demographic and cardiometabolic parameters. To attenuate these major confounders, we widely adjusted for central adiposity and LDL-c, and, in sensitivity analyses, insulin resistance. Even so, the magnitude of these baseline differences raises the possibility of residual confounding, which cannot be fully excluded in an observational design. Nevertheless, the persistence of the associations with large/very large TRL-p and TRL mean size after wide adjustment and sensitivity analyses suggests they are unlikely to be entirely attributable to these baseline differences. Additionally, we did not apply formal correction for multiple comparisons across TRL-p subclasses and TSA90 categorizations; our findings should therefore be interpreted as hypothesis-strengthening rather than definitive, and replication in independent cohorts is warranted. On the other hand, strengths include the large ELSA-Brasil sample size, NMR precision for TRL subclass determination, comprehensive multivariate adjustment, and formal statistical testing of the nonlinear dose–response through RCS regression. To our knowledge, this is the first study assessing TRL-p subclasses in sleep disorders, filling a critical gap.

In conclusion, larger TRL-p are independently associated with mild and moderate OSA, even after extensive multivariate adjustments for confounders. Moreover, even brief episodes of nocturnal hypoxia are correlated with increased levels in these TRL subclasses, which might contribute to atherogenic dyslipidemia.

## Data availability

The data underlying this study cannot be made publicly available due to ethical restrictions approved by the research ethics committees of the participating institutions and by the Publications Committee of ELSA-Brasil (publiELSA), as they contain potentially identifying and sensitive participant information. De-identified data can be made available to qualified researchers upon reasonable request and submission of a research proposal to the ELSA-Brasil Datacenter (estatistica@elsa.ufrgs.br) and the ELSA-Brasil Publications Committee, subject to their approval.

## Supplemental data

This article contains supplemental data.

## Conflicts of interest

R. D. S. is a consultant for AstraZeneca, Daiichi Sankyo, Eli Lilly, Esperion, MSD, Novartis, Novo Nordisk, and Ultragenyx. He has received speaker honoraria from Amgen, Novo Nordisk, Novartis, Eli Lilly, and Daiichi Sankyo. He has participated in trials run by Amgen, Arrowhead, AstraZeneca, Ionis, MSD, Novartis, and Sanofi/Regeneron. L. F. D. is a consultant and speaker for ResMed. All other authors declare that they have no conflicts of interest with the contents of this article.
