# Supplementary material for: Impact of sleep apnea severity and nocturnal hypoxemia on the triglyceride-rich lipoproteins particles: the ELSA-Brasil study
Source: J Lipid Res. 2026 Jul 14;67(8):101104. doi: 10.1016/j.jlr.2026.101104 (PMC13487270; doi:10.1016/j.jlr.2026.101104)
Supplement: Supplementary file 1 [file mmc1.docx]

**Supplementary Material**

Table S1 – Lipid profile according to peripheral oxygen saturation classification

| % Time under SpO_2_ < 90% | No hypoxemia | Tertile 1 | tertile 2 | tertile 3 | p 0vs1 | p 0vs2 | p 0vs3 | p 1vs3 | p 2vs3 |
| --- | --- | --- | --- | --- | --- | --- | --- | --- | --- |
| LDL-p (nmol/L) | 1530.7 ± 407.9 | 1609.8 ± 416.2 | 1690.3 ± 422.5 | 1686.0 ± 418.9 | 0.004 | <0.001 | <0.001 | 0.014 | 1.000 |
| HDL-p (µmol/L) | 21.85 ± 2.72 | 21.97 ± 2.95 | 22.32 ± 2.87 | 22.27 ± 3.15 | 1.000 | 0.029 | 0.065 | 0.510 | 1.000 |
| TRL-p size (nm) | 43.91 ± 8.15 | 45.89 ± 8.70 | 47.77 ± 8.99 | 49.21 ± 9.55 | <0.001 | <0.001 | <0.001 | <0.001 | 0.039 |
| TRL-P total (nmol/L) | 133.45 ± 65.94 | 148.63 ± 75.82 | 155.58 ± 72.72 | 162.99 ± 75.53 | 0.001 | <0.001 | <0.001 | 0.006 | 0.527 |
| TRL-c | 29.30 ± 10.89 | 31.45 ± 12.03 | 33.41 ± 12.14 | 33.97 ± 12.87 | 0.009 | <0.001 | <0.001 | 0.003 | 1.000 |
| LDL-p size (nm) | 20.901 ± 0.544 | 20.780 ± 0.615 | 20.710 ± 0.610 | 20.654 ± 0.611 | 0.002 | <0.001 | <0.001 | 0.003 | 0.700 |
| HDL-p size (nm) | 9.000 ± 0.403 | 8.897 ± 0.391 | 8.822 ± 0.351 | 8.814 ± 0.375 | <0.001 | <0.001 | <0.001 | 0.002 | 1.000 |
| APO B (mg/dL) | 88.70 ± 22.97 | 93.29 ± 23.88 | 97.25 ± 23.65 | 97.15 ± 23.97 | 0.003 | <0.001 | <0.001 | 0.040 | 1.000 |
| Apo A-1 (mg/dL) | 139.73 ± 23.48 | 137.18 ± 23.16 | 137.10 ± 22.88 | 136.79 ± 22.60 | 0.294 | 0.253 | 0.144 | 1.000 | 1.000 |
| VAP HDL-c ratio | 0.384 ± 0.109 | 0.359 ± 0.098 | 0.345 ± 0.092 | 0.339 ± 0.097 | <0.001 | <0.001 | <0.001 | 0.007 | 1.000 |
| LDL-p large (%) | 43.20 ± 18.55 | 43.96 ± 18.69 | 45.18 ± 18.66 | 43.38 ± 18.45 | 1.000 | 0.363 | 1.000 | 1.000 | 0.668 |
| LDL-p small (%) | 50.91 ± 24.11 | 56.39 ± 25.79 | 60.64 ± 27.12 | 60.61 ± 25.80 | 0.001 | <0.001 | <0.001 | 0.039 | 1.000 |

Table S2 - Linear regression analysis according to AHI cutoff (no OSA/mild vs. moderate/severe)

|  | AHI | Crude β (95% CI) | Model 1 β (95% CI) | Model 2 β (95% CI) | Model 3 β (95% CI) |
| --- | --- | --- | --- | --- | --- |
| TRL-cholesterol (mg/dL) | <15 | Reference | | | |
|  | ≥15 | 4.74 (3.57; 5.91) | 3.26 (2.07; 4.46) | 2.62 (1.42; 3.82) | 0.65 (-0.50; 1.80) |
| TRL-P (nmol/L) | <15 | Reference | | | |
|  | ≥15 | 21.90 (15.77; 28.03) | 14.77 (8.47; 21.07) | 12.22 (5.83; 18.60) | 2.49 (-3.40; 8.37) |
| Large TRL-P (nmol/L) | <15 | Reference | | | |
|  | ≥15 | 2.17 (1.64; 2.69) | 1.70 (1.16; 2.25) | 1.22 (0.68; 1.76) | 0.27 (-0.28; 0.83) |
| Medium TRL-P (nmol/L) | <15 | Reference | | | |
|  | ≥15 | 4.63 (3.11; 6.15) | 2.94 (1.41; 4.48) | 2.26 (0.71; 3.81) | 0.91 (-0.71; 2.54) |
| Small/very small TRL-P (nmol/L) | <15 | Reference | | | |
|  | ≥15 | 15.11 (9.97; 20.25) | 10.13 (4.81; 15.44) | 8.75 (3.34; 14.15) | 1.31 (-3.47; 6.08) |
| TRL mean  size (nm) | <15 | Reference | | | |
|  | ≥15 | 3.80 (3.05; 4.56) | 3.20 (2.43; 3.98) | 2.45 (1.67; 3.22) | 0.67 (-0.10; 1.45) |

Table S3 – Association between nocturnal hypoxemia (rate of sleep time under SpO₂ <90%, TSA90, in tertiles) and triglyceride-rich lipoprotein subclasses and mean size in participants not receiving antidiabetic treatment (n = 2,208), Model 3 with additional adjustment for ln(HOMA-IR).

| TSA90 | β (95% CI) | P value |
| --- | --- | --- |
| Large/very large TRL-p (nmol/L) |  |  |
| Zero | Reference |  |
| Tertile 1 | 0.48 (-0.14; 1.09) | 0.127 |
| Tertile 2 | 0.60 (-0.07; 1.27) | 0.081 |
| Tertile 3 | 0.80 (0.06; 1.54) | 0.034 |
| P for trend | 0.26 (0.03; 0.50) | 0.029 |
| ln(HOMA-IR) | 1.66 (1.37; 1.96) | <0.001 |
| TRL mean size (nm) |  |  |
| Zero | Reference |  |
| Tertile 1 | 0.72 (-0.17; 1.61) | 0.115 |
| Tertile 2 | 0.99 (0.03; 1.95) | 0.043 |
| Tertile 3 | 1.13 (0.05; 2.21) | 0.040 |
| P for trend | 0.38 (0.04; 0.73) | 0.028 |
| ln(HOMA-IR) | 2.40 (1.97; 2.83) | <0.001 |
| Medium TRL-p (nmol/L) |  |  |
| Zero | Reference |  |
| Tertile 1 | 1.52 (-0.32; 3.37) | 0.106 |
| Tertile 2 | 1.43 (-0.65; 3.51) | 0.178 |
| Tertile 3 | -0.32 (-2.55; 1.92) | 0.780 |
| P for trend | -0.01 (-0.73; 0.70) | 0.970 |
| ln(HOMA-IR) | 3.65 (2.75; 4.55) | <0.001 |

*β coefficients (95% CI) from linear regression with van der Waerden–transformed outcomes. Reference = no nocturnal hypoxemia (TSA90 = 0). TSA90 tertile cutoffs: tertile 1 (0.1–0.5%), tertile 2 (0.6–3.1%), tertile 3 (≥ 3.2%). Model 3 was adjusted for age, sex, race, smoking status, hypertension, LDL-c, and waist circumference, plus ln(HOMA-IR); the diabetes term was removed to avoid collinearity with HOMA-IR. P for trend was obtained by modeling the tertile rank as a continuous score. HOMA-IR, homeostatic model assessment of insulin resistance; TRL, triglyceride-rich lipoprotein.*

Table S4 – Association between nocturnal hypoxemia (rate of sleep time under SpO₂ <90%, TSA90, in tertiles) and triglyceride-rich lipoprotein subclasses and mean size in participants receiving antidiabetic treatment (n = 164), Model 3 with fasting plasma glucose in place of HOMA-IR.

| TSA90 | β (95% CI) | P value |
| --- | --- | --- |
| Large/very large TRL-p (nmol/L) |  |  |
| Zero | Reference |  |
| Tertile 1 | -0.90 (-3.69; 1.88) | 0.524 |
| Tertile 2 | 3.66 (-0.50; 7.82) | 0.084 |
| Tertile 3 | 1.07 (-2.09; 4.23) | 0.504 |
| Fasting plasma glucose | 0.030 (0.003; 0.057) | 0.030 |
| TRL mean size (nm) |  |  |
| Zero | Reference |  |
| Tertile 1 | -1.18 (-5.36; 2.99) | 0.576 |
| Tertile 2 | 1.98 (-2.34; 6.29) | 0.367 |
| Tertile 3 | 1.22 (-2.74; 5.17) | 0.545 |
| Fasting plasma glucose | 0.046 (0.011; 0.082) | 0.011 |
| Medium TRL-p (nmol/L) |  |  |
| Zero | Reference |  |
| Tertile 1 | -1.67 (-10.09; 6.76) | 0.696 |
| Tertile 2 | 9.34 (0.58; 18.10) | 0.037 |
| Tertile 3 | 2.87 (-4.82; 10.57) | 0.462 |
| Fasting plasma glucose | 0.034 (-0.017; 0.086) | 0.191 |

*β coefficients (95% CI) from linear regression with van der Waerden–transformed outcomes. Reference = no nocturnal hypoxemia (TSA90 = 0). TSA90 tertile cutoffs as in Supplementary Table S1. Model 3 was adjusted for age, sex, race, smoking status, hypertension, LDL-c, and waist circumference, with fasting plasma glucose substituted for HOMA-IR, which is not interpretable under glucose-lowering therapy. This subgroup is small and underpowered; estimates are exploratory and shown for completeness. TRL, triglyceride-rich lipoprotein.*

Table S5 - Association between nocturnal hypoxemia (rate of sleep time under SpO₂ <90%, TSA90, in tertiles) and triglyceride-rich lipoprotein measures, with the diabetes diagnosis replaced by continuous glycated hemoglobin.

| **TSA90** | **β (95% CI)** | **P value** |
| --- | --- | --- |
| **Large/very large TRL-p (nmol/L)** | | |
| No nocturnal hypoxemia (TSA90 = 0) | Reference | — |
| Tertile 1 | 0.38 (−0.22 to 0.99) | 0.216 |
| Tertile 2 | 0.84 (0.16 to 1.52) | 0.015 |
| Tertile 3 | 0.77 (0.04 to 1.51) | 0.039 |
| *P for trend* | — | *0.017* |
| ln(HbA1c) | 4.63 (2.10 to 7.17) | <0.001 |
| **Medium TRL-p (nmol/L)** | | |
| No nocturnal hypoxemia (TSA90 = 0) | Reference | — |
| Tertile 1 | 1.44 (−0.38 to 3.27) | 0.122 |
| Tertile 2 | 2.21 (0.17 to 4.24) | 0.033 |
| Tertile 3 | 0.21 (−1.98 to 2.40) | 0.849 |
| *P for trend* | — | *0.563* |
| ln(HbA1c) | 8.14 (1.75 to 14.53) | 0.013 |
| **TRL mean size (nm)** | | |
| No nocturnal hypoxemia (TSA90 = 0) | Reference | — |
| Tertile 1 | 0.68 (−0.21 to 1.58) | 0.132 |
| Tertile 2 | 1.20 (0.25 to 2.16) | 0.013 |
| Tertile 3 | 1.15 (0.08 to 2.22) | 0.035 |
| *P for trend* | — | *0.018* |
| ln(HbA1c) | 4.27 (1.42 to 7.12) | 0.003 |
|  | | |

*Values are linear regression β coefficients (95% CI). Models were fully adjusted (Model 3: age, sex, race, hypertension, smoking status, LDL-c, and waist circumference), with the binary diabetes term replaced by the natural logarithm of glycated hemoglobin to avoid collinearity (n = 2,371). The reference group is participants without nocturnal hypoxemia (TSA90 = 0). P for trend was obtained by entering the tertile rank as a continuous score. TSA90, percentage of sleep time with SpO₂ <90%; HbA1c, glycated hemoglobin; LDL-c, low-density lipoprotein cholesterol; TRL, triglyceride-rich lipoprotein; TRL-p, TRL particle number; CI, confidence interval.*

Table S6 - Association between nocturnal hypoxemia (rate of sleep time under SpO₂ <90%, TSA90, in tertiles) and triglyceride-rich lipoprotein measures in participants not using lipid-lowering drugs (n = 2,107).

| **TSA90 tertiles** | **β (95% CI)** | **P-value** |
| --- | --- | --- |
| **Large/very large TRL-p (nmol/L)** |  |  |
| Tertile 0 (reference) | Reference |  |
| Tertile 1 | 0.42 (-0.21; 1.05) | 0.195 |
| Tertile 2 | 0.72 (0.03; 1.41) | 0.041 |
| Tertile 3 | 0.70 (-0.06; 1.45) | 0.071 |
| **Medium TRL-p (nmol/L)** |  |  |
| Tertile 0 (reference) | Reference |  |
| Tertile 1 | 1.46 (-0.47; 3.38) | 0.137 |
| Tertile 2 | 1.20 (-0.98; 3.37) | 0.280 |
| Tertile 3 | -0.88 (-3.14; 1.37) | 0.443 |
| **TRL mean size (nm)** |  |  |
| Tertile 0 (reference) | Reference |  |
| Tertile 1 | 0.72 (-0.20; 1.65) | 0.125 |
| Tertile 2 | 1.21 (0.21; 2.22) | 0.018 |
| Tertile 3 | 1.21 (0.05; 2.36) | 0.041 |

*Values are linear regression β coefficients (95% CI). Models were fully adjusted (Model 3: age, sex, race, hypertension, smoking status, LDL-c, diabetes diagnosis and waist circumference). The reference group is participants without nocturnal hypoxemia (TSA90 = 0). LDL-c, low-density lipoprotein cholesterol; TRL, triglyceride-rich lipoprotein; TRL-p, TRL particle number; CI, confidence interval.*

Table S7 – Association between nocturnal hypoxemia (rate of sleep time under SpO₂ <90%, TSA90, in tertiles) and triglyceride-rich lipoprotein measures in participants not using beta-blockers or thiazide diuretics (n = 1,952).

| **TSA90 tertiles** | **β (95% CI)** | **P value** |
| --- | --- | --- |
| **Large/very large TRL-p (nmol/L)** | | |
| Tertile 0 (reference) | Reference | — |
| Tertile 1 | 0.53 (−0.11 to 1.18) | 0.106 |
| Tertile 2 | 0.90 (0.20 to 1.60) | 0.011 |
| Tertile 3 | 0.89 (0.12 to 1.65) | 0.024 |
| **Medium TRL-p (nmol/L)** | | |
| Tertile 0 (reference) | Reference | — |
| Tertile 1 | 1.88 (−0.06 to 3.82) | 0.058 |
| Tertile 2 | 1.96 (−0.28 to 4.20) | 0.087 |
| Tertile 3 | −0.54 (−2.82 to 1.74) | 0.640 |
| **TRL mean size (nm)** | | |
| Tertile 0 (reference) | Reference | — |
| Tertile 1 | 0.76 (−0.19 to 1.71) | 0.115 |
| Tertile 2 | 1.35 (0.30 to 2.39) | 0.011 |
| Tertile 3 | 1.10 (−0.06 to 2.25) | 0.062 |

*Values are linear regression β coefficients (95% CI). Models were fully adjusted (Model 3: age, sex, race, smoking status, hypertension, diabetes, LDL-c, and waist circumference). The reference group is participants without nocturnal hypoxemia (TSA90 = 0). TSA90 tertile cutoffs as in Supplementary Table S1. Analysis restricted to participants not using beta-blockers or thiazide diuretics (n = 1,952). LDL-c, low-density lipoprotein cholesterol; TRL, triglyceride-rich lipoprotein; TRL-p, TRL particle number; CI, confidence interval.*

Table S8. Baseline characteristics according to obstructive sleep apnea severity (AHI <15 vs ≥15 events/h), stratified by sex.

|  | Men | | | | Women | | | |
| --- | --- | --- | --- | --- | --- | --- | --- | --- |
| Characteristic | Total  (n=1,040) | AHI <15  (n=582) | AHI ≥15  (n=458) | P | Total  (n=1,357) | AHI <15  (n=1,013) | AHI ≥15  (n=344) | P |
| Age (years) | 49.4 (8.5) | 48.3 (8.3) | 50.7 (8.6) | <0.001 | 49.3 (8.2) | 48.2 (7.8) | 52.8 (8.3) | <0.001 |
| Race/ethnicity, n (%) |  |  |  | 0.044 |  |  |  | 0.95 |
| Black | 126 (12.2) | 77 (13.3) | 49 (10.9) |  | 165 (12.2) | 124 (12.4) | 41 (12.0) |  |
| Mixed Brown | 245 (23.8) | 128 (22.1) | 117 (26.0) |  | 244 (18.1) | 181 (18.0) | 63 (18.4) |  |
| White | 608 (59.1) | 354 (61.1) | 254 (56.4) |  | 847 (62.9) | 632 (62.9) | 215 (62.7) |  |
| Asian | 38 (3.7) | 14 (2.4) | 24 (5.3) |  | 84 (6.2) | 61 (6.1) | 23 (6.7) |  |
| Indigenous | 12 (1.2) | 6 (1.0) | 6 (1.3) |  | 7 (0.5) | 6 (0.6) | 1 (0.3) |  |
| Smoking status, n (%) |  |  |  | 0.093 |  |  |  | 0.33 |
| Never | 523 (50.2) | 307 (52.7) | 216 (47.1) |  | 809 (59.6) | 607 (59.9) | 202 (58.7) |  |
| Former | 362 (34.8) | 186 (32.0) | 176 (38.3) |  | 363 (26.8) | 262 (25.9) | 101 (29.4) |  |
| Current | 156 (15.0) | 89 (15.3) | 67 (14.6) |  | 185 (13.6) | 144 (14.2) | 41 (11.9) |  |
| Hypertension, n (%) | 340 (32.7) | 130 (22.3) | 210 (45.8) | <0.001 | 303 (22.3) | 183 (18.1) | 120 (34.9) | <0.001 |
| Diabetes mellitus, n (%) | 195 (18.7) | 73 (12.5) | 122 (26.6) | <0.001 | 189 (13.9) | 114 (11.3) | 75 (21.8) | <0.001 |
| Insulin (µUI/mL) | 6.7 (3.9–11.2) | 5.5 (2.9–9.1) | 8.3 (5.3–13.6) | <0.001 | 6.1 (3.7–10.2) | 5.5 (3.3–9.4) | 7.9 (5.2–12.6) | <0.001 |
| HOMA-IR | 1.80 (1.01–3.11) | 1.46 (0.81–2.51) | 2.40 (1.40–3.77) | <0.001 | 1.55 (0.90–2.67) | 1.41 (0.81–2.45) | 2.12 (1.28–3.34) | <0.001 |
| Body mass index (kg/m²) | 27.0 (4.3) | 25.5 (3.6) | 28.9 (4.5) | <0.001 | 27.1 (5.0) | 26.1 (4.5) | 30.0 (5.5) | <0.001 |
| Waist circumference (cm) | 93.2 (11.4) | 89.1 (9.9) | 98.3 (11.0) | <0.001 | 85.8 (12.0) | 83.4 (11.0) | 92.9 (12.1) | <0.001 |
| Fasting plasma glucose (mg/dL) | 112.1 (25.9) | 108.0 (18.7) | 117.4 (32.1) | <0.001 | 105.5 (20.7) | 103.8 (18.1) | 110.6 (26.3) | <0.001 |
| Total cholesterol (mg/dL) | 212.0 (40.8) | 208.1 (37.0) | 216.9 (44.7) | <0.001 | 211.3 (38.0) | 209.2 (37.2) | 217.5 (39.8) | <0.001 |
| Triglycerides (mg/dL) | 129.0 (92.0–188.0) | 120.0 (83.0–171.0) | 141.0 (101.0–201.0) | <0.001 | 100.0 (73.0–143.0) | 95.0 (70.0–136.0) | 115.5 (88.0–162.0) | <0.001 |
| HDL-cholesterol (mg/dL) | 50.7 (12.1) | 50.8 (11.6) | 50.5 (12.8) | 0.64 | 59.9 (13.8) | 60.3 (13.7) | 58.7 (14.0) | 0.064 |
| LDL-cholesterol (mg/dL) | 130.9 (33.4) | 129.4 (31.2) | 132.9 (36.0) | 0.091 | 128.2 (32.6) | 126.8 (32.2) | 132.3 (33.5) | 0.006 |
| Apolipoprotein B (mg/dL) | 96.2 (24.3) | 93.7 (23.0) | 99.3 (25.4) | <0.001 | 91.7 (23.4) | 90.0 (22.9) | 96.8 (24.0) | <0.001 |
| Apolipoprotein A-1 (mg/dL) | 131.5 (21.2) | 131.1 (20.1) | 132.0 (22.6) | 0.54 | 142.7 (23.3) | 143.0 (23.0) | 141.9 (24.1) | 0.46 |
| Total TRL-p (nmol/L) | 164.5 (73.3) | 159.1 (67.6) | 171.4 (79.6) | 0.008 | 136.7 (70.4) | 131.3 (68.0) | 152.7 (74.9) | <0.001 |
| Large/very large TRL-p (nmol/L) | 2.9 (0.3–8.1) | 1.8 (0.2–6.7) | 4.4 (1.0–9.5) | <0.001 | 1.0 (0.1–4.5) | 0.7 (0.1–4.0) | 2.6 (0.4–6.7) | <0.001 |
| Medium TRL-p (nmol/L) | 26.6 (20.6) | 25.6 (20.0) | 27.8 (21.3) | 0.088 | 16.8 (14.4) | 16.0 (13.6) | 19.2 (16.2) | <0.001 |
| Small/very small TRL-p (nmol/L) | 132.2 (61.0) | 128.7 (54.3) | 136.7 (68.5) | 0.035 | 116.7 (60.1) | 112.4 (58.5) | 129.1 (63.1) | <0.001 |

*Data are presented as mean (SD), median (IQR), or n (%). Groups are defined by the apnea–hypopnea index (AHI): <15 vs ≥15 events/h. P values were obtained from Student’s t test (normally distributed continuous variables), the Mann–Whitney U test (skewed continuous variables), or the χ² test (categorical variables), as appropriate.*

*AHI, apnea–hypopnea index; HDL, high-density lipoprotein; HOMA-IR, homeostatic model assessment of insulin resistance; LDL, low-density lipoprotein; TRL-p, triglyceride-rich lipoprotein particles.*

Table S9 – Association between nocturnal hypoxemia (rate of sleep time under SpO₂ <90%, TSA90, in tertiles) and triglyceride-rich lipoprotein measures stratified by sex.

| **TSA90** | **Men (n=1,040)**  **β (95% CI)** | **Women (n=1,357)**  **β (95% CI)** |
| --- | --- | --- |
| **Large/very large TRL-p (nmol/L)** |  |  |
| Tertile 0 (reference) | — | — |
| Tertile 1 | 0.34 (−0.82; 1.50) | 0.34 (−0.30; 0.98) |
| Tertile 2 | 1.11 (−0.16; 2.38) | 0.65 (−0.07; 1.37) |
| Tertile 3 | 1.16 (−0.21; 2.52) | 0.26 (−0.49; 1.01) |
| **Medium TRL-p (nmol/L)** |  |  |
| Tertile 0 (reference) | — | — |
| Tertile 1 | 1.32 (−2.21; 4.84) | 1.00 (−0.94; 2.94) |
| Tertile 2 | 1.56 (−2.18; 5.29) | 2.32 (0.04; 4.59)* |
| Tertile 3 | −0.75 (−4.60; 3.10) | 0.40 (−2.08; 2.88) |
| **TRL mean size (nm)** |  |  |
| Tertile 0 (reference) | — | — |
| Tertile 1 | 0.58 (−0.98; 2.15) | 0.62 (−0.44; 1.69) |
| Tertile 2 | 1.45 (−0.20; 3.09) | 0.94 (−0.20; 2.08) |
| Tertile 3 | 0.91 (−0.83; 2.65) | 1.17 (−0.21; 2.54) |

Table S10 - Decomposition of the attenuation of the association between nocturnal hypoxemia (time with SpO_2_ <90%, TSA90, tertiles) and triglyceride-rich lipoprotein particle measures: effect of waist circumference and LDL-C, added separately, on Model 2.

| **TSA90** | **Model 2** | **Model 2 + LDL-C** | **Model 2 + WC** | **Full Model 3** |
| --- | --- | --- | --- | --- |
|  | ***β (95% CI)*** | ***β (95% CI)*** | ***β (95% CI)*** | ***β (95% CI)*** |
| **Large/very large TRL-P (nmol/L)** |  |  |  |  |
| Zero | *Reference* | | |  |
| Tertile 1 | 0.78 (0.12; 1.45) | 0.81 (0.19; 1.43) | 0.38 (-0.22; 0.99) | 0.41 (-0.25; 1.06) |
| Tertile 2 | 1.82 (1.14; 2.50) | 1.86 (1.19; 2.53) | 0.88 (0.19; 1.56) | 0.92 (0.23; 1.60) |
| Tertile 3 | 2.19 (1.49; 2.90) | 2.20 (1.49; 2.91) | 0.81 (0.08; 1.55) | 0.80 (0.07; 1.54) |
| **Medium TRL-P (nmol/L)** |  |  |  |  |
| Zero | *Reference* | | |  |
| Tertile 1 | 2.13 (0.21; 4.04) | 1.99 (0.17; 3.82) | 1.57 (-0.27; 3.40) | 1.47 (-0.45; 3.38) |
| Tertile 2 | 3.79 (1.84; 5.73) | 3.57 (1.61; 5.53) | 2.47 (0.41; 4.52) | 2.32 (0.32; 4.33) |
| Tertile 3 | 2.34 (0.32; 4.36) | 2.09 (0.05; 4.14) | 0.40 (-1.79; 2.59) | 0.25 (-1.90; 2.40) |
| **TRL mean size (nm)** |  |  |  |  |
| Zero | *Reference* | | |  |
| Tertile 1 | 1.39 (0.44; 2.35) | 1.49 (0.56; 2.43) | 0.61 (-0.28; 1.51) | 0.71 (-0.21; 1.62) |
| Tertile 2 | 2.97 (2.00; 3.94) | 3.14 (2.18; 4.10) | 1.13 (0.18; 2.09) | 1.27 (0.32; 2.23) |
| Tertile 3 | 3.81 (2.81; 4.82) | 3.93 (2.88; 4.97) | 1.12 (0.04; 2.20) | 1.17 (0.15; 2.20) |

*Values are linear regression β coefficients (95% CI). CI, confidence interval; LDL-C, low-density lipoprotein cholesterol; TRL-P, triglyceride-rich lipoprotein particles; TSA90, time with peripheral oxygen saturation below 90%; WC, waist circumference.*

*Model 2 was adjusted for age, sex, race/skin color, smoking status, hypertension, and diabetes. “Model 2 + WC” adds waist circumference alone; “Model 2 + LDL-C” adds LDL-C alone; Model 3 adds both waist circumference and LDL-C. Reference category: no nocturnal hypoxemia (TSA90 = 0).*
